# Arachidonate 15-Lipoxygenase Type B Knockdown Leads to Reduced Lipid Accumulation and Inflammation in Atherosclerosis

**DOI:** 10.1371/journal.pone.0043142

**Published:** 2012-08-17

**Authors:** Lisa U. Magnusson, Annika Lundqvist, Merja Nurkkala Karlsson, Kristina Skålén, Max Levin, Olov Wiklund, Jan Borén, Lillemor Mattsson Hultén

**Affiliations:** Sahlgrenska Center for Cardiovascular and Metabolic Research, Wallenberg Laboratory, Department of Molecular and Clinical Medicine, Institute of Medicine, Sahlgrenska Academy, University of Gothenburg, Gothenburg, Sweden; Heart Center Munich, Germany

## Abstract

Inflammation in the vascular wall is important for development of atherosclerosis. We have shown previously that arachidonate 15-lipoxygenase type B (ALOX15B) is more highly expressed in human atherosclerotic lesions than in healthy arteries. This enzyme oxidizes fatty acids to substances that promote local inflammation and is expressed in lipid-loaded macrophages (foam cells) present in the atherosclerotic lesions. Here, we investigated the role of ALOX15B in foam cell formation in human primary macrophages and found that silencing of human *ALOX15B* decreased cellular lipid accumulation as well as proinflammatory cytokine secretion from macrophages. To investigate the role of ALOX15B in promoting the development of atherosclerosis *in vivo,* we used lentiviral shRNA silencing and bone marrow transplantation to knockdown mouse *Alox15b* gene expression in LDL-receptor-deficient (*Ldlr* ^−/−^) mice. Knockdown of mouse *Alox15b in vivo* decreased plaque lipid content and markers of inflammation. In summary, we have shown that ALOX15B influences progression of atherosclerosis, indicating that this enzyme has an active proatherogenic role.

## Introduction

Atherosclerosis is a complex disease that involves chronic inflammation at every stage from initiation to progression and eventually to plaque rupture [1]. Macrophage-derived foam cells play integral roles in atherosclerosis and are key regulators of the lipid-driven proinflammatory responses that promote atherosclerosis [2]. An inflammatory subset of macrophages accumulates in atherosclerotic plaques and produces proinflammatory cytokines [2]–[3] as well as lipoxygenases, which oxygenate polyunsaturated fatty acids to proinflammatory mediators [4]. Arachidonate 15-lipoxygenase (ALOX15) catalyzes the production of eicosanoids that activate monocyte integrins and thereby enhance the adhesion of monocytes to the endothelium [5]–[6]. *In vitro*, ALOX15 appears to be proatherogenic, as it can be directly involved in LDL oxidation [7]–[9]. *In vivo,* however, both pro- and anti-atherogenic effects have been reported (reviewed in [10]). For example, atherosclerotic lesion formation is increased in *Ldlr* ^−/−^ mice that overexpress human ALOX15 in the endothelium [11]. In contrast, transgenic rabbits overexpressing human ALOX15 in macrophages display reduced atherosclerotic lesion area in the thoracic aorta [12].

Previous studies of lipoxygenases in the development of atherosclerotic plaques have largely focused on ALOX15 type A (ALOX15A) [13]. However, ALOX15 type B (ALOX15B) is expressed at considerably higher levels than ALOX15A in human carotid plaque macrophages [14]–[15] and levels of ALOX15B have been shown to be higher in symptomatic compared with asymptomatic lesions [14] suggesting a role for this enzyme in the atherosclerotic process. Furthermore, we have previously shown that overexpression of ALOX15B in macrophages increases secretion of the chemokines CXCL10 and CCL2, which are chemoattractants for T cells and monocytes [16]. In addition, products released in response to increased ALOX15B activation lead to increased expression of the T-cell activation marker CD69 as well as increased T-cell migration [16].

Here, we investigated the role of ALOX15B in foam cell formation in human monocyte-derived macrophages and found that silencing of the gene encoding ALOX15B (*ALOX15B*) decreases cellular lipid accumulation as well as proinflammatory signaling from macrophages. We also investigated the role of the mouse ortholog of ALOX15B (encoded by the gene *Alox15b*, also known as *Alox8* [17]) in *Ldlr* ^−/−^ mice. In contrast to human ALOX15B, which catalyzes the formation of 15(S)-hydroperoxy eicosatetraenoic acid (HPETE), mouse ALOX15B produces 8(S)-HPETE and 8(S)-, 15(S)-diHPETE from arachidonic acid. However, the two enzymes display the highest sequence identity among all the mammalian lipoxygenases and substitution of only two amino acids of mouse ALOX15B is required to change the eicosanoids generated to 90% 15S-HETE [18]. We observed that mice transplanted with mouse *Alox15b* knockdown bone marrow displayed decreased atherosclerosis and impaired immune signaling. On the basis of our results, we suggest that ALOX15B has a proatherogenic and proinflammatory role during atherogenesis.

## Materials and Methods

### Primary macrophages

Buffy coats were obtained from healthy adult volunteer blood donors at Kungälv Hospital, Sweden, and samples were de-identified before handling. Human mononuclear cells were isolated by centrifugation in a discontinuous gradient of Ficoll-Paque (GE Healthcare). Cells were seeded in Macrophage-SFM medium (Gibco) containing granulocyte macrophage colony stimulating factor (GM-CSF). After 3 days, the medium was changed to RPMI medium without GM-CSF and cells were cultured for 7 days before transfection. Macrophages were transfected with 20 nmol/L *ALOX15B* siRNA (Qiagen, SI03076206) or nonsilencing control siRNA (Qiagen, 1027280) in HiPerFect transfection reagent (Qiagen) according to the manufacturer's recommendations. Cells were washed after 24 h, siRNA was added again and cells were incubated with or without dimethyloxalylglycine (DMOG) for 24 h before extraction of RNA. DMOG is an inhibitor of HIF-prolyl hydroxylase, and acts to stabilize HIF-1α expression causing induction of *ALOX15B* [15].

### Mice

C57BL/6 *Ldlr* ^−/−^ mice (7-week-old males) were purchased from The Jackson Laboratory (Bar Harbor, ME, USA) and housed in a pathogen-free barrier facility. Bone marrow donor mice of the same genotype (13-week-old males) were also purchased. All animal studies were approved by the regional animal ethics committee (animal ethics committee of Gothenburg).

### *Alox15b*-silenced mice

Silencing of *Alox15b* in mice of C57BL/6 *Ldlr* ^−/−^ background was accomplished *in vivo* through lentiviral short-hairpin (sh)RNA silencing [19] using shRNA delivered with lentivirus to bone marrow from donor mice. Lentiviral transduction particles containing *Alox15b* shRNA (TRCN0000076452, NM_009661.2-700s1c1) or nonsilencing control shRNA (SHC002) respectively were purchased from Sigma. Bone marrow including hematopoetic stemcells with genotype *Ldlr* ^−/−^ were isolated from C57Bl/6 *Ldlr* ^−/−^ mice and infected with either *Alox15b* shRNA encoding or control shRNA encoding lentivirus over night in Stem span media (Stemcell technologies). Cells were harvested and injected retroorbitally to lethally irradiated (9 Gy) recipient mice. The mice were fed a western type diet (Harlan TD88137) for 20 weeks.

### Analysis of gene and protein expression

Total RNA was isolated with the RNeasy kit (Qiagen). Expression of human *ALOX15B* and mouse *Alox15b* mRNA was determined and normalized to β-actin mRNA expression using quantitative real-time PCR. The reverse transcription reaction was set up using a cDNA reverse transcription kit (#4368814) and performed with a Gene Amp PCR system 9700 (Applied Biosystems). Real time PCR amplification was set up using Taq man gene expression assays for human *ALOX15A* (Hs00609608_m1), human *ALOX15B* (Hs00153988_m1), mouse *Alox15b* (Mm01325281_m1), human *ACTB* (Hs99999903_m1) mouse *Emr1* (Mm00802529_m1) and mouse *ActB* (Mm00607939_s1) respectively in combination with Universal PCR master mix (#4324018) and performed for 50 cycles on an ABI PRISM 7700 sequence detection system (Applied Biosystems).

Immunoblots were prepared as described [20]. Total cellular lysates were prepared from human macrophages, mouse bone marrow macrophages and aortic tissue. Antibodies are listed in Table 1. The polyclonal ALOX15B antibody (LX25) has been well characterized and found to detect human as well as mouse ALOX15B but not ALOX15A [21].

**Table 1:** Antibodies used in this study.

	Source	Antibody	Description	2^nd^ antibody
**ALOX15B**	Oxford^1^	LX25	Rabbit, poly^4^	Goat anti rabbit HRP^6^ GE^7^
**ALOX15A**	Abcam	ab54772	Mouse, mono^5^	Goat anti mouse HRP^6^ GE^7^
**CD4** (T cells)	BD^2^	550278	Rat, mono^5^	Goat anti rat HRP^6^, GE^7^
**CD8** (T cells)	BD^2^	550281	Rat, mono^5^	Goat anti rat HRP^6^, GE^7^
**Mac-2**	NB^3^	CL8942AP	Rat	Goat anti rat HRP^6^, GE^7^
**α-actin** Cy3	Sigma	C6198	Mouse, mono^5^	**-**

1 Oxford Biochemical research,

2 BD Bioscience,

3 Nordic biosite,

4 Polyclonal,

5 Monoclonal,

6 Horse rabbit peroxidase,

7 GE healthcare.

### Quantification of Oil Red O-stained lipid droplets

Human macrophages were grown on chamber slides (Lab-Tek Systems), stained with Oil Red O and hematoxylin, and viewed with a Zeiss Axioplan 2 microscope. Twenty images per slide were obtained and analyzed for red pixels (lipid droplets), cell size and cell number using BioPix iQ 2.2.1 (Gothenburg, Sweden, see www.biopix.se for further information) [22].

### Cytokine analysis

Secreted cytokines [interferon (IFN)-γ, interleukin (IL)-1β, IL-6, IL-8 and IL-12 (total)] were analyzed in medium from cultured primary human macrophages using Human Proinflammatory Multiplex assay (Meso Scale Discovery; K15007C) according to the manufacturer's instructions and a SECTOR Imager 2400 reader (Meso Scale Discovery).

Mouse plasma IL-2 was analyzed using a Mouse TH1/TH2 Multiplex assay (Meso Scale Discovery; K15013C) according to the manufacturer's instructions and a SECTOR Imager 2400 reader (Meso Scale Discovery).

### Quantification of atherosclerosis

The aortic roots were embedded in OCT Tissue-Tec medium and immediately frozen in liquid nitrogen. Cryostat sections (10 µm thick) were cut from the level of the aortic valves and distally, and stained in 0.5% Oil Red O or used for immunohistochemistry (see next section). Distal aortae were dissected free from adipose and connective tissue, cut open longitudinally, and pinned flat on silicone-coated dishes. The aorta was then fixed in 70% ethanol for 5 minutes, stained with 0.5% Sudan IV for 6 minutes, and differentiated for 3 minutes in 80% ethanol. Digital images of the entire vessel were captured and the outline of the aortic surface was defined manually, whereas the stained lesion area was defined using computerized color selection in BioPix iQ 2.2.1 (Gothenburg, Sweden). The extent of atherosclerosis was calculated as the percentage of the aortic surface covered by lesions.

### Immunohistochemistry

Cryostat sections of the aortic root were fixed for 10 minutes in ice-cold acetone, dried, and blocked in 5% goat serum in Tris-buffered saline. Sections were incubated (60 minutes, room temperature) with antibodies according to Table 1. Endogenous peroxidase was silenced with 0.3% H_2_O_2_ in H_2_O for 10 minutes. 3-amino-9-ethylcarbazole (AEC) was used for detection. α-actin was stained using a Cy3 conjugated primary antibody. Collagen was stained using Masson's trichrome stains according to the manufacturer's instructions (Sigma; HT-15). Pictures were taking using a Zeiss Axioplan 2 epifluorescence microscope with an Axiocam camera and Axiovision software. Quantification of stained lesion area was defined using computerized color selection in BioPix iQ 2.2.1 (Gothenburg, Sweden, see www.biopix.se for further information) [22].

## Results

### Knockdown of *ALOX15B* in human macrophages decreases cellular lipid levels, foam cell size and cytokine secretion

**Figure 1:**
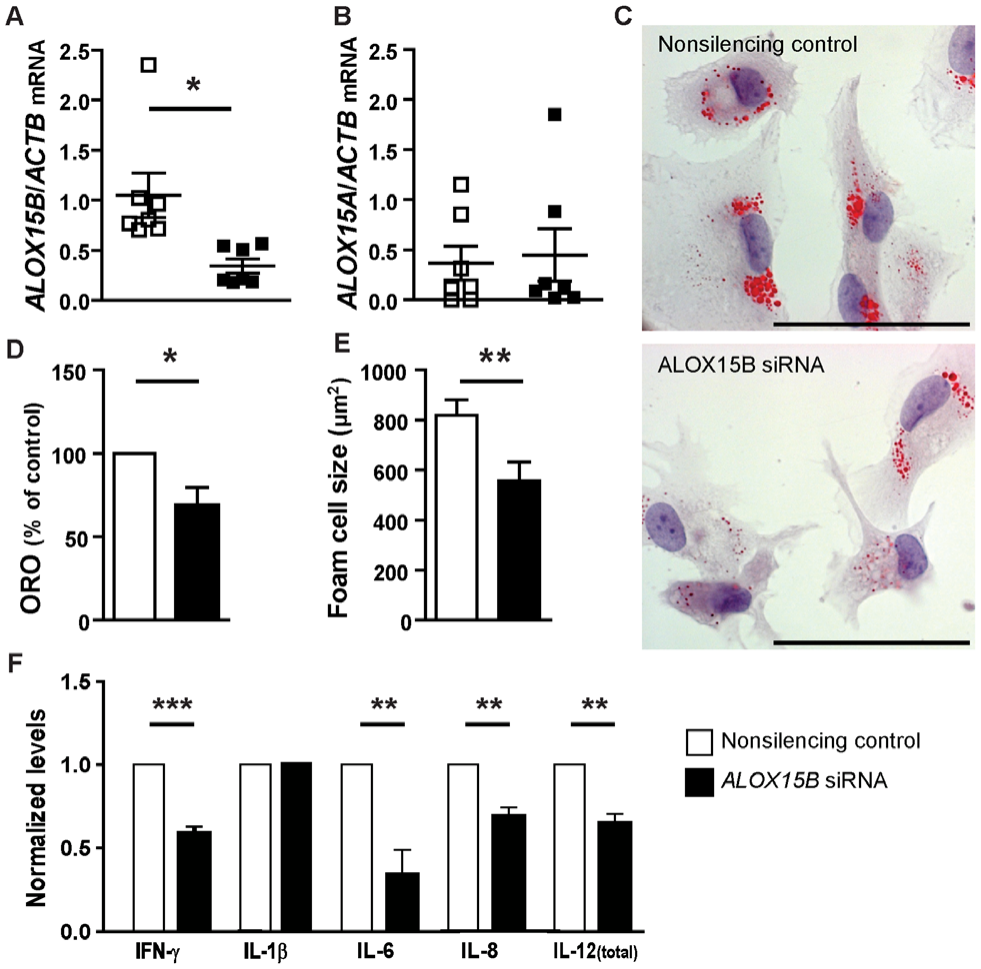
Decreased lipid uptake and immunological signaling in human *ALOX15B*-silenced macrophages. Lipid accumulation was analyzed in human primary macrophages transfected with nonsilencing control siRNA or *ALOX15B* siRNA using Oil Red O staining after incubation with DMOG. **A**) Quantification of *ALOX15B* expression normalized to *ActB* expression measured with Q-PCR. **B**) Quantification of *ALOX15A* expression normalized to *ActB* expression measured with Q-PCR. **C**) Representative picture showing Oil Red O staining of control and *ALOX15B*-silenced macrophages (Scale bar = 50 µm). **D**) Quantification of Oil Red O staining in human primary macrophages (n = 7) normalized to control. **E**) Size of control and *ALOX15B*-silenced human primary macrophages (foam cells). **F**) Quantification of secreted cytokines in media from human primary macrophages (n = 7). Data are presented as mean±SEM normalized to control.

Transfection of human primary macrophages with *ALOX15B* siRNA resulted in knockdown of *ALOX15B* mRNA to approximately 50% of nonsilenced levels (**Fig. 1A**), but did not affect the expression of *ALOX15A* mRNA (**Fig. 1B**) or ALOX15A protein (too low levels to detect, data not shown). *ALOX15B* knockdown resulted in decreased lipid droplet accumulation as shown by reduced Oil Red O staining (**Fig. 1C and D**) and decreased foam cell size (**Fig. 1E**). These data indicate that ALOX15B can promote lipid droplet formation. *In vitro* experiments with human primary macrophages also showed lower levels of secreted IFN-γ, IL-6, IL-8 and IL-12 (total) while there was no change in the levels of IL-1β (**Fig. 1F**) in the medium when *ALOX15B* was knocked down using siRNA, indicating that ALOX15B affects secretion of some proinflammatory cytokines.

### Knockdown of mouse *Alox15b* decreases atherosclerosis in mice aorta

**Figure 2:**
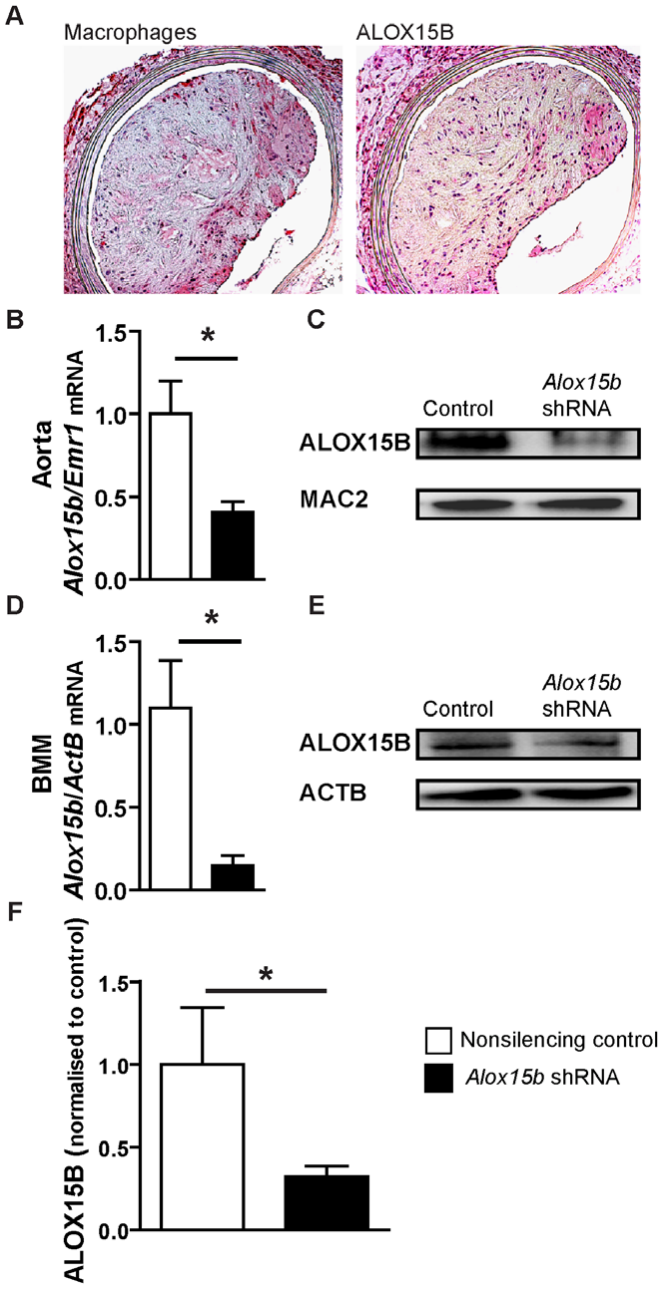
*Alox15b* knockdown in *LDLr^−/−^* mice. **A**) Immunohistochemical detection of macrophages and ALOX15B in *Ldlr^−/−^* mice. **B**) Quantification of *Alox15b* expression, normalized to *Emr1* expression (macrophage marker) in aortic tissue using Q-PCR (n = 2 for control and n = 3 for *Alox15b* shRNA). The sections presented in figure 2A were stained with Mayer's hematoxylin while the quantified sections used for figure 2B were not. **C**) Western blotting of ALOX15B and MAC-2 (macrophage marker) in aortic tissue. **D**) Quantification of *Alox15b* expression in bone marrow macrophages (BMM) isolated and differentiated at the end of the silencing experiment using Q-PCR (n = 2 for control and n = 3 for *Alox15b* shRNA). **E**) Western blotting of ALOX15B and ACTB in bone marrow macrophages. **F**) ALOX15B levels measured by immunohistochemistry in sections from aortic sinus from control and *Alox15b* knockdown mice (n = 7 per group). Data are presented as mean±SEM.

Immunohistochemical analysis of serial sections of aorta from cholesterol-fed *Ldlr* ^−/−^ mice showed that mouse ALOX15B was expressed in macrophage-rich areas of atherosclerotic plaques in mice (**Fig. 2A**).

To investigate the importance of mouse ALOX15B in atherogenesis, lethally irradiated *Ldlr* ^−/−^ mice were transplanted with bone marrow cells infected with lentivirus containing *Alox15b* shRNA or nonsilencing control shRNA and fed a western diet for 20 weeks to induce atherosclerosis. Knockdown of *Alox15b* was confirmed by Q-PCR of mRNA and western blotting of protein from aortic tissue (**Fig. 2B and C**) and bone marrow macrophages (**Fig. 2D and E**) from recipient mice and by immunohistochemical evaluation of the proximal aorta from recipient mice (**Fig. 2F**).

**Figure 3:**
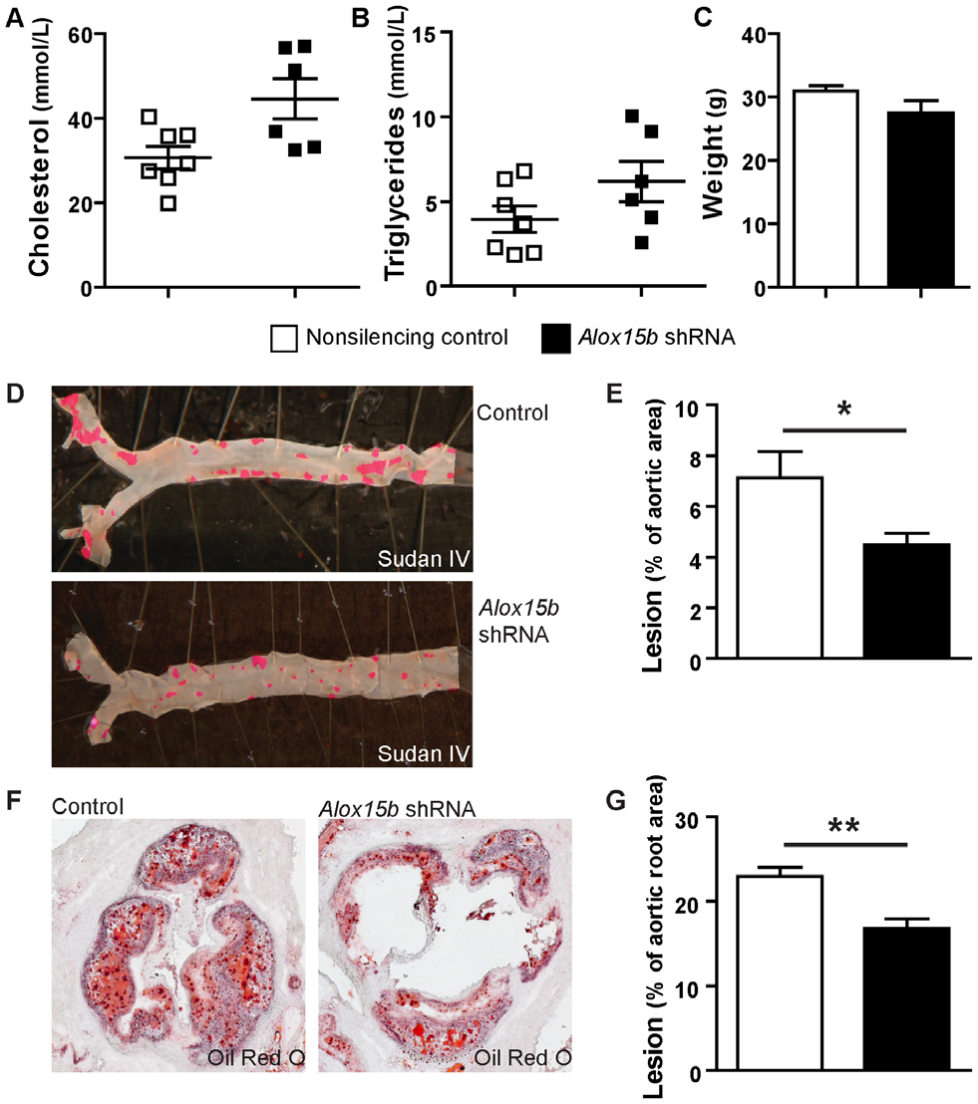
Decreased atherosclerotic lesions in aortas in *Alox15b* knockdown mice. **A**) Plasma cholesterol, **B**) plasma triglycerides and **C**) body weight of *Alox15b* knockdown and control mice. **D**) Representative photographs showing aorta pinned out by en face technique and stained with Sudan IV. **E**) Quantification of subendothelial lipid accumulation in the aorta (n = 7 per group). **F**) Representative histological analysis of the aortic sinus stained with Oil Red O. **G**) Quantification of subendothelial lipid accumulation in the aortic root (n = 6 per group). Data are presented as mean±SEM.

Body weight, plasma cholesterol and triglycerides did not differ between the two groups (**Fig. 3A, B, C**). Atherosclerotic lesions both in the whole aorta (**Fig. 3D and E**) and in the aortic root (**Fig. 3F and G**) were significantly decreased in mice that received *Alox15b* knockdown bone marrow compared to nonsilencing control.

These data show that atherosclerotic lesions are decreased in *Alox15b* knockdown mice.

### Knockdown of mouse *Alox15b* reduces T cell number and IL-2 levels *in vivo*

**Figure 4:**
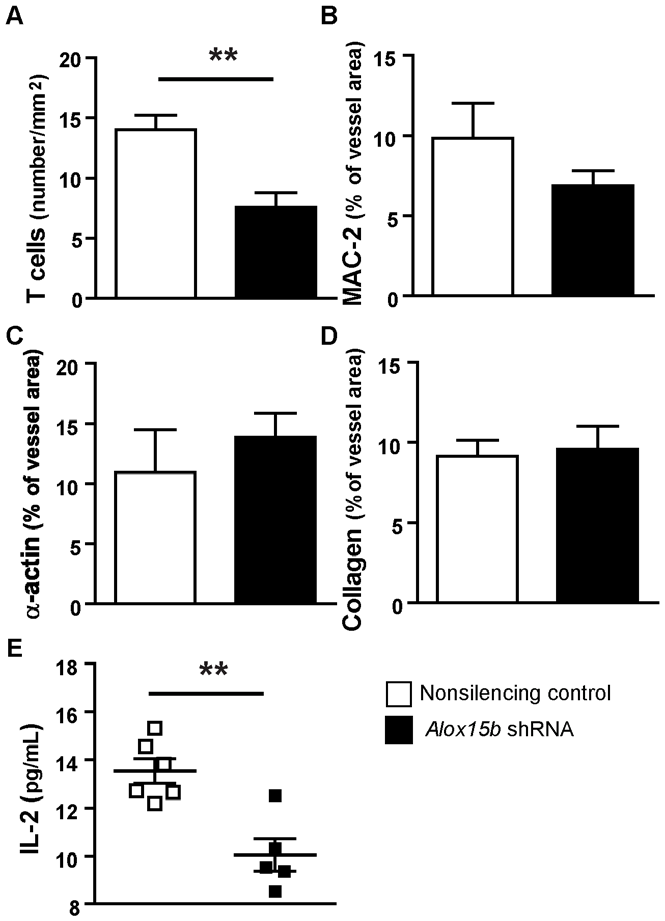
Analysis of plaque composition and plasma levels of IL-2. Sections from aortic sinuses were stained with antibodies against **A**) CD4/CD8 (T cells), **B**) MAC-2 (macrophages), **C**) α-actin (smooth muscle cells), and **D**) collagen. Data are presented as mean±SEM. **E**) Plasma levels of soluble IL-2. (n = 6 per group).

The number of T cells was significantly reduced in lesion areas in *Alox15b* knockdown mice (**Fig. 4A**) while no significant change was found in plaque macrophage content (**Fig. 4B**). Markers of plaque stability (collagen and α-actin) showed no change (**Fig. 4C and D**). To evaluate the possible proinflammatory role of mouse ALOX15B, we also measured cytokine levels in plasma from *Alox15b* knockdown mice compared to nonsilencing shRNA control-animals. The knockdown animals displayed significantly reduced plasma levels of IL-2 (**Fig. 4E**) indicating that *Alox15b* knockdown may cause decreased systemic inflammation.

## Discussion

In the present report, we studied the effects of decreased ALOX15B levels in human macrophages as well as in an atherosclerotic mouse model. Silencing of *ALOX15B* in human macrophages decreased cellular lipid accumulation and reduced proinflammatory cytokine secretion. *In vivo* experiments in an atherosclerotic mouse model showed that mouse *Alox15b* knockdown resulted in decreased atherosclerosis (measured as plaque area) as well as decreased inflammation, providing new evidence for ALOX15B as a proatherogenic protein.

*ALOX15B* knockdown in human macrophages caused markedly decreased cellular lipid accumulation and foam cell size, suggesting that ALOX15B is involved in lipid accumulation and foam cell formation. Silencing of *ALOX15B* decreased secretion of the proinflammatory cytokines IFN-γ IL-6, IL-8 and IL-12 from the macrophages. IFN-γ is known to promote foam cell formation [2] suggesting that the anti-foam cell formation effect of *ALOX15B* knockdown could be partly due to lower levels of IFN-γ. Previous data show that ALOX15B expression in human macrophages induces chemokine secretion and that the preconditioned medium from these macrophages positively affects T-cell migration [23].

To understand the role of ALOX15B in atherosclerosis *in vivo*, we studied the consequence of *Alox15b* knockdown in an atherosclerotic mouse model. Although mouse ALOX15B differs from human ALOX15B, the two enzymes display 78% sequence identity and overproduction of human or mouse ALOX15B inhibited growth in mouse keratinocytes to the same extent, suggesting common signaling pathways [24]. Upon knockdown of *Alox15b* in the atherosclerotic mouse model, we found that subendothelial lipid accumulation was decreased in aortas from *Ldlr* ^−/−^ mice that received *Alox15b* knockdown bone marrow. The difference in lipid accumulation was not due to differences in plaque macrophage content, as no significant change in MAC-2 staining between the two groups was detected. Since silencing of human *ALOX15B* affected lipid accumulation in human macrophages, it is reasonable to propose that the effect on subendothelial lipid accumulation in mouse plaques is at least partly due to direct effects on foam cell formation.

Plaque T-cell content and plasma levels of IL-2 were significantly decreased in mice that received *Alox15b*-deficient bone marrow, indicating that mouse ALOX15B may also affect immunoregulation. Systemic administration of IL-2 to *ApoE^−/−^* mice has been shown to cause increased atherogenesis while anti-IL-2 antibodies decreased development of atherosclerosis [25]. IL-2 plays a significant role in T-cell activation and proliferation [26]. In agreement, our previous results show that human ALOX15B overexpression in human macrophages induces activation and migration of T cells [16]. Decreased T-cell content as well as decreased IL-2 levels in mice that received *Alox15B* deficient bone marrow are consistent with a role for mouse ALOX15B and its products in recruiting T cells to the lesion. Lesional T cells mainly have properties of the proinflammatory Th1 subtype and secrete the cytokines IL-2, IFN-γ, and tumor necrosis factor (TNF)-α. These cytokines cause activation of macrophages and vascular cells, promote inflammation and also participate in cellular immunity [27]. Atherosclerosis is a Th1-cell-driven disease and Th1 cytokines stimulate plaque formation [28]. Furthermore IL-2 promotes angiogenesis by activation of Akt (protein kinase B) and increase of reactive oxygen species [29]. Angiogenesis in human lesions may be maladaptive as it promotes lesion instability, with increased risk of plaque rupture and cardiovascular events [30]. It is likely that the reduced T-cell content in plaques of mice that received *Alox15b*-deficient bone marrow is caused by decreased immunological signaling from plaque macrophages, since silencing of human *ALOX15B* caused reduced cytokine expression in human macrophages.

Our experiments show that reduction of ALOX15B decreases inflammation and lipid accumulation, both in human primary macrophages and in mice, suggesting an active proinflammatory and proatherogenic role of ALOX15B.
